# Naive CD8^+^ T-cell precursors display structured TCR repertoires and composite antigen-driven selection dynamics

**DOI:** 10.1038/icb.2015.17

**Published:** 2015-03-24

**Authors:** Michelle A Neller, Kristin Ladell, James E McLaren, Katherine K Matthews, Emma Gostick, Johanne M Pentier, Garry Dolton, Andrea JA Schauenburg, Dan Koning, Ana Isabel CA Fontaine Costa, Thomas S Watkins, Vanessa Venturi, Corey Smith, Rajiv Khanna, Kelly Miners, Mathew Clement, Linda Wooldridge, David K Cole, Debbie van Baarle, Andrew K Sewell, Scott R Burrows, David A Price, John J Miles

**Affiliations:** 1 Human Immunity Laboratory, Cellular Immunology Laboratory and Tumour Immunology Laboratory, Queensland Institute of Medical Research, Brisbane, QLD, Australia; 2 Institute of Infection and Immunity, Cardiff University School of Medicine, Heath Park, Cardiff, UK; 3 Department of Immunology, University Medical Center Utrecht, Utrecht, The Netherlands; 4 Computational Biology Unit, Centre for Vascular Research, University of New South Wales, Kensington, NSW, Australia; 5 School of Medicine, The University of Queensland, Brisbane, QLD, Australia; 6 Human Immunology Section, Vaccine Research Center, National Institute of Allergy and Infectious Diseases, National Institutes of Health, Bethesda, MD, USA

## Abstract

Basic parameters of the naive antigen (Ag)-specific T-cell repertoire in humans remain poorly defined. Systematic characterization of this ‘ground state' immunity in comparison with memory will allow a better understanding of clonal selection during immune challenge. Here, we used high-definition cell isolation from umbilical cord blood samples to establish the baseline frequency, phenotype and T-cell antigen receptor (TCR) repertoire of CD8^+^ T-cell precursor populations specific for a range of viral and self-derived Ags. Across the board, these precursor populations were phenotypically naive and occurred with hierarchical frequencies clustered by Ag specificity. The corresponding patterns of TCR architecture were highly ordered and displayed partial overlap with adult memory, indicating biased structuring of the T-cell repertoire during Ag-driven selection. Collectively, these results provide new insights into the complex nature and dynamics of the naive T-cell compartment.

Reactive T cells from the extrathymic naive pool are expanded and mobilized into the memory repertoire by specific and productive interactions with cognate antigen (Ag). Exactly which clonotypes are recruited during this process, however, remains a source of recurrent immunological inquiry. At present, we understand that extrathymic αβ T-cell selection is dependent on a number of variables, including Ag abundance, priming location, T-cell antigen receptor (TCR) ligand binding parameters and precursor frequency (reviewed in Allen *et al.*^1^).

Recent technical advances in the detection and isolation of T-cell precursors directly *ex vivo* have allowed, for the first time, the unambiguous enumeration and characterization of unmanipulated naive Ag-specific populations.^2^ Initial experiments in mice revealed that Ag-specific precursors are present at frequencies of 0.08–890 cells per 100 000 CD4^+^/CD8^+^ T cells (reviewed in Jenkins and Moon^3^). In addition, T-cell precursor frequencies were found to cluster numerically by Ag specificity between different mice. Interestingly, early precursor enumerations appeared to correlate positively with immunodominance hierarchies after pathogen challenge.^3^ However, recent evidence in other murine systems^4, 5^ demonstrated that this association can sometimes be inverse, indicating that memory formation is complex and involves the proliferative capabilities of individual T-cell precursors. In humans, initial calculations from adult peripheral blood place Ag-specific precursor frequencies between 2 and 600 cells per 100 000 CD4^+^/CD8^+^ T cells.^6, 7, 8, 9, 10^

In this study, we aimed to determine several baseline parameters of Ag-specific precursors in humans through the use of umbilical cord blood (UCB). We combined a modified multimer-based magnetic enrichment protocol with high-definition multiparametric flow cytometry to ensure the high-purity isolation, accurate enumeration and detailed phenotypic characterization of Ag-specific precursors directly *ex vivo*. Furthermore, we ascertained TCR usage in these Ag-specific precursor populations by using a quantitative molecular approach and conducted a systematic comparison with our large bank of memory TCR repertoires from adults to explore the effects of Ag-driven selection. This study represents the first enumeration and analysis of Ag-specific CD8^+^ T-cell precursors in human newborns.

## Results

### Sample selection and high-definition T-cell isolation

We used UCB to characterize the baseline Ag-specific T-cell precursor repertoire in order to bypass certain technical limitations seen when using adult material. First, in adults, identification of authentic naive cells is sometimes difficult due to subsets of memory T cells that exhibit 'naive-like' phenotypes.^11^ Second, naive T cells steadily proliferate in adults over time, which can distort precursor calculations.^12^ Third, adult humans have lengthy and multifaceted immune histories, such that the naive/memory T-cell ratio can vary widely between individuals.^12^ This variable can drastically alter precursor estimates when calculating frequencies across total lymphocytes or lymphocyte subsets. Finally, over the long duration of a human life, it is difficult to rule out contact with any given infectious agent or immunogenic compound. This is particularly applicable to ubiquitous influenza viruses and human herpesviruses, which can produce false negatives or discrepant results during serotesting.^13, 14^ In contrast, the large bulk of the UCB compartment is naive and, in healthy mothers, vertical transmission of the pathogens investigated here is rare.

**Figure 1:**
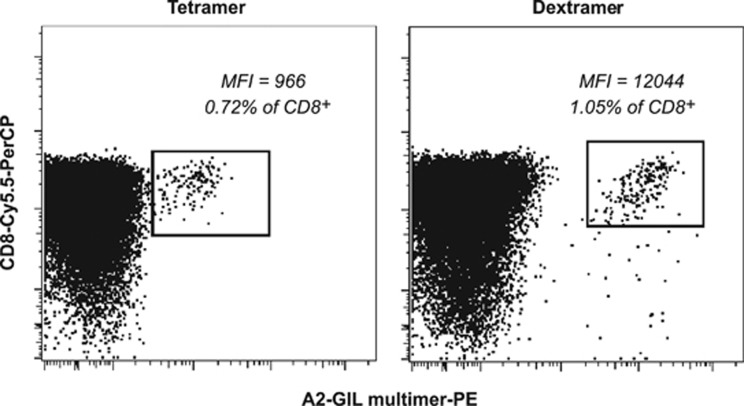
Comparison of multimers for use in magnetic enrichment. An HLA-A2^+^ UCB sample was divided and labeled with PE-conjugated A2-GIL tetramer or dextramer, then magnetically enriched using αPE beads. Enriched cells were surface stained for CD8 expression and acquired using a FACSCanto II flow cytometer. The gating strategy is shown in Figure 2. Numbers indicate the percentage of multimer^+^ cells within the enriched CD8^+^ population. The median fluorescence intensity of each multimer^+^ population is shown.

To ensure high-definition isolation of low-frequency T cells, we first compared tetramers versus dextramers, which comprise long dextran polymer backbones individually decorated with ~24 peptide-major histocompatibility complex and ~12 fluorochrome molecules,^15^ for their ability to identify naive precursors in UCB. At the optimal median fluorescence intensity titration for each reagent, dextramers proved superior to tetramers in parallel multiparametric flow cytometry trials (Figure 1). Specifically, dextramer-labeled populations were more readily identifiable and >12-fold brighter than their tetramer-labeled counterparts. These findings concur with a recent study in which dextramers consistently outperformed tetramers, most strikingly for the identification of low-avidity T-cell populations.^16^ To further facilitate precursor isolation, we combined dextramers with dasatinib pre-treatment, which can enhance peptide-major histocompatibility complex multimer fluorescence without concomitant increases in background staining by blocking Ag-induced TCR downregulation.^17^ As predicted, we observed brighter staining of Ag-specific precursors in the presence of dasatinib (data not shown). It is noteworthy that this approach does not alter T-cell phenotype.^16^ Accordingly, all multimer magnetic enrichments in this study were performed by using a combination of dasatinib pre-treatment and dextramer staining.

### Analysis of naive Ag-specific T-cell precursor frequencies

**Figure 2:**
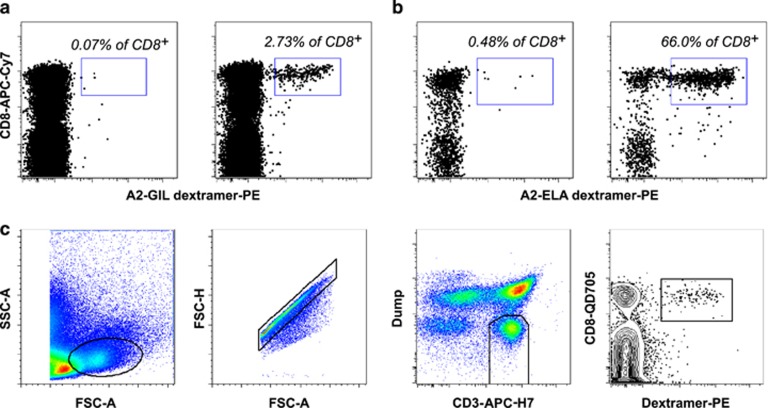
Magnetic enrichment and gating strategy for naive Ag-specific T-cell precursor isolation. (**a** and **b**) UCB samples were compared before and after magnetic enrichment for A2-GIL (**a**) and A2-ELA (**b**). Dextramer^+^ cells were either undetectable or barely detectable in unenriched UCB. In contrast, dextramer^+^ populations were clearly visible after magnetic enrichment. Numbers indicate the percentage of dextramer^+^ cells within the enriched CD8^+^ population. (**c**) Gating strategy used for high-definition flow cytometric sorting. The dump channel comprised LIVE/DEAD Fixable Violet Dead Cell Stain together with αCD14 and αCD19 mAbs conjugated to Pacific Blue. Single lymphocytes were selected based on light scatter properties. Non-dump CD3^+^ cells were gated and Ag-specific T cells were identified in a CD8 versus dextramer bivariate plot.

**Figure 3:**
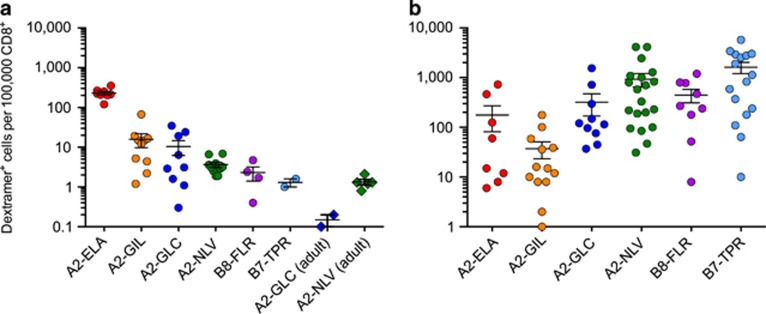
*Ex vivo* enumeration of Ag-specific precursors and memory T cells from humans. (**a**) The number of dextramer^+^ cells per 100  000 CD8^+^ cells was calculated from 46 UCB samples and seven herpesvirus-seronegative adult peripheral blood mononuclear cell (PBMC) samples. (**b**) The number of dextramer^+^ cells per 100,000 CD8^+^ cells was calculated from 72 herpesvirus-seropositive adult PBMC samples. Ag-specific precursor enumeration was achieved via dextramer magnetic enrichment with the exception of A2-ELA-specific cells, which were detectable in unmanipulated UCB. The flow cytometric gating strategy is shown in Figure 2c. Statistically significant differences were identified between Ag specificities (Supplementary Table 2). Among precursor populations, A2-ELA-specific T cells were significantly more frequent compared with all other Ag specificities (*P*<0.001). Among memory populations, A2-GIL-specific T cells were significantly less frequent compared with other viral specificities (*P*<0.05).

Six different dextramers were synthesized bearing a series of viral and self-derived Ags as follows: HLA-A*0201-ELAGIGILTV (A2-ELA) from human Melan-A/MART-1 (residues 26–35), HLA-A*0201-GILGFVFTL (A2-GIL) from influenza virus matrix (residues 58–66), HLA-A*0201-GLCTLVAML (A2-GLC) from Epstein–Barr virus (EBV) BMLF1 (residues 280–288), HLA-A*0201-NLVPMVATV (A2-NLV) from cytomegalovirus (CMV) pp65 (residues 495–503), HLA-B*0801-FLRGRAYGL (B8-FLR) from EBV EBNA3A (residues 325–333) and HLA-B*0702-TPRVTGGGAM (B7-TPR) from CMV pp65 (residues 417–426). Using these reagents in conjunction with a modified magnetic enrichment protocol (Figures 2a and b) and a strict flow cytometric gating strategy (Figure 2c), we observed clustering of T-cell precursor frequencies based around epitope specificity (Figure 3a and Supplementary Table 1). A2-ELA-specific precursors were by far the most frequent in the UCB repertoire, averaging 229 cells per 100 000 CD8^+^ cells (range 120–352). Virus-specific precursors were rarer by more than an order of magnitude in all cases, with a hierarchy per 100 000 CD8^+^ cells as follows: A2-GIL-specific precursors, mean 15.8 cells (range 1.2–66.9)>A2-GLC-specific precursors, mean 10.4 cells (range 0.3–34.7)>A2-NLV-specific precursors, mean 3.7 cells (range 1.9–6.9)>B8-FLR-specific precursors, mean 2.3 cells (range 0.4–4.7)>B7-TPR-specific precursors, mean 1 cell (range <1–1.6). In contrast, the immunodominance hierarchy in virus-exposed adults was more or less inverted, with memory T-cells specific for the herpesviruses EBV and CMV dominating the CD8^+^ compartment (Figure 3b). A2-GLC-specific precursors were detected at a mean of 0.15 cells per 100 000 CD8^+^ cells (range 0.1–0.2) in EBV-seronegative adults and A2-NLV-specific precursors were detected at a mean of 1.3 cells per 100 000 CD8^+^ cells (range 0.8–2.1) in CMV-seronegative adults (Figure 3a and Supplementary Table 1). These numbers are substantially lower than the corresponding frequencies in UCB samples but comparable with those reported previously in adults.^3^ Statistical analysis revealed highly significant differences between precursor frequencies across Ag specificities in UCB, most notably for A2-ELA in all comparisons with viral specificities (Supplementary Table 2A). A more nuanced picture emerged in adults, although A2-GIL-specific precursors occurred at consistently lower frequencies compared with other viral specificities (Supplementary Table 2B). Collectively, these data demonstrate that precursor frequencies cluster by Ag specificity and vary with age.

### Phenotypic analysis of naive Ag-specific T-cell precursors

**Figure 4:**
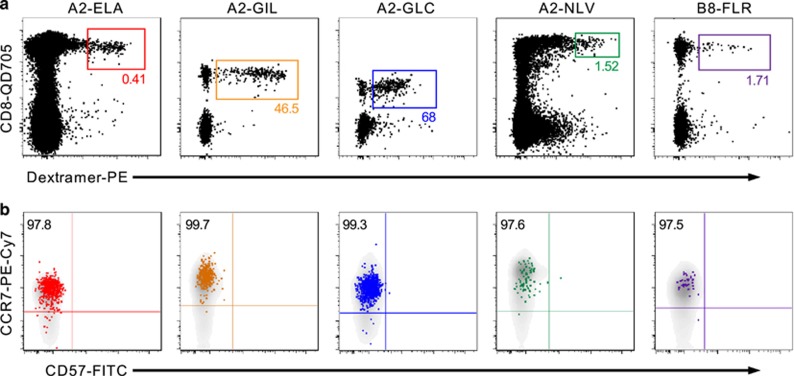
*Ex vivo* phenotyping of naive Ag-specific T-cell precursors. (**a**) Representative flow cytometry plots showing sort gates for naive T-cell populations across five epitope specificities as indicated. Ag-specific T cells were identified via dextramer magnetic enrichment with the exception of A2-ELA-specific cells, which were detectable in unmanipulated UCB. Numbers indicate the percentage of dextramer^+^ cells within the total CD8^+^ population. (**b**) The phenotype of dextramer^+^ cells (coloured) overlaid on all CD3^+^ T cells (gray), showing CCR7 expression and the absence of CD57. Numbers indicate the percentage of dextramer^+^ cells expressing this naive phenotype. Other surface marker analyses yielded similar data.

Multiparametric flow cytometry-based phenotypic data were acquired using an optimized panel built around ten distinct fluorochromes. The core gating strategy and representative analyses of dextramer^+^ T-cell precursor populations are shown in Figures 2 and 4, respectively. For all epitope specificities across all UCB samples, dextramer^+^ cells displayed the classic naive T-cell phenotype (CD27^hi^ CD45RA^hi^ CD45RO^lo^ CD57^lo^ CCR7^hi^). These data help to verify the truly naive status of Ag-specific T-cell precursors identified in the present study.

### Clonotypic analysis of naive Ag-specific T-cell precursors

**Figure 5:**
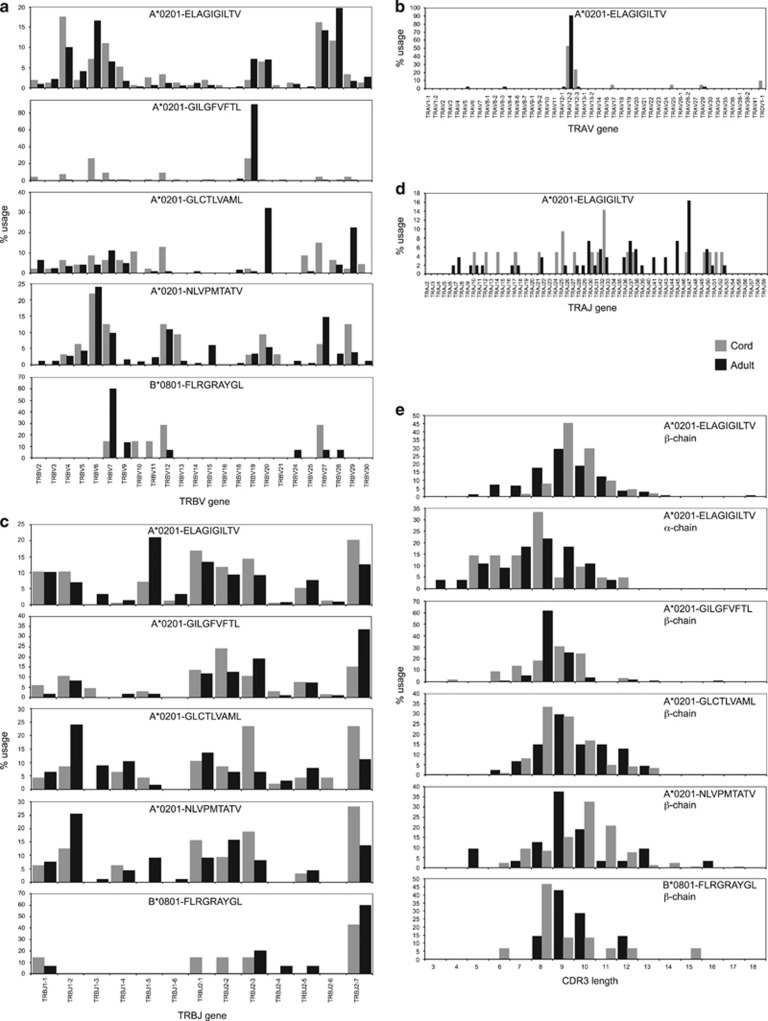
Comparison of *ex vivo* Ag-specific TCR repertoires between naive and memory T cells. (**a**–**e**) *TRBV* gene usage (**a**), *TRAV* gene usage (**b**), *TRBJ* gene usage (**c**), *TRAJ* gene usage (**d**) and CDR3 length calculated using the Chothia nomenclature (**e**). Individual clonotypes were weighted by appearance with each unique sequence counted once regardless of frequency. The database comprised 7880 sequences derived from 85 Ag-specific TCR repertoires. Naive and memory sequences, respectively, were compared for A2-ELA (612 and 2134), A2-GIL (144 and 631), A2-GLC (145 and 1127), A2-NLV (245 and 2330) and B8-FLR (174 and 338).

Next, we examined TCR usage in naive Ag-specific T-cell precursor populations by sorting magnetically enriched dextramer^+^ cells at >98% purity directly into microtubes containing an RNA protectant and using a template-switch anchored PCR with reverse transcription to amplify all expressed *TR* gene transcripts without bias.^18^ Final cell numbers varied between 30 and 2000 per sample depending on epitope specificity and population frequency. To contextualize the data, we compared precursor TCR transcripts (1320 sequences) with our bank of adult memory TCR transcripts covering the same specificities (6550 sequences). The similarities and differences in TCR gene usage and CDR3 length between naive precursors and memory cells are illustrated in Figure 5.

For A2-ELA-specific T cells, *TRBV* and *TRBJ* gene usage showed considerable overlap and some minor divergence (Figures 5a and c). In particular, *TRBV4* and *TRBJ2-7* were enriched in UCB, while *TRBV6* and *TRBJ1-5* were enriched in adults. The CDR3β lengths showed Gaussian distributions, with dual preference for a loop length of nine residues (Figure 5e). As A2-ELA-specific T cells in adults are known to have biased TCR α-chain architecture,^19^ we examined *TRA* gene transcripts in the corresponding UCB precursor populations. Like adults, naive precursors were heavily biased toward *TRAV12* gene usage (Figure 5b); *TRAJ* gene usage was not especially ordered between populations (Figure 5d).

For A2-GIL-specific T cells, *TRBV* and *TRBJ* gene usage also showed some commonalities between adults and UCB with bias toward *TRBV19* (Figure 5a). Interestingly, in UCB precursors but not adult cells, *TRBV* gene usage was evenly split between *TRBV6-5* and *TRBV19*. Both cohorts showed enrichment for *TRBJ2* genes, with adults displaying a strong preference for *TRBJ2-7* (Figure 5c). The CDR3β lengths in both populations were distributed in a Gaussian fashion with adult memory cells more focused on a peak loop length of eight residues (Figure 5e).

For A2-GLC-specific T cells, *TRBV* gene usage was broad across both naive and memory populations with moderate overlap (Figure 5a). Adults displayed a preference for *TRBV20-1* and *TRBV29-1*, while *TRBV12* and *TRBV27* were preferred in UCB. A similar pattern applied to *TRBJ* gene usage, with moderate overlap and population-specific bias (Figure 5c). Adults displayed a preference for *TRBJ1-2*, while UCB precursors preferentially used either *TRBJ2-3* or *TRBJ2-7*. The CDR3β lengths were superimposable between populations (Figure 5e).

For A2-NLV-specific T cells, *TRBV* gene usage was broad across both naive and memory populations with considerable overlap (Figure 5a). However, UCB precursors were enriched for *TRBV29-1* and adult memory cells were enriched for *TRBV27*. A similar pattern applied to *TRBJ* gene usage, with broad overlap and enrichment for *TRBJ2-7* and *TRBJ1-2* in UCB and adults, respectively (Figure 5c). The CDR3β lengths in both populations were distributed in a Gaussian fashion, although UCB precursors preferred a longer loop length of ten residues (Figure 5e).

For B8-FLR-specific T cells, *TRBV* gene usage was noticeably biased in both UCB precursors and adults (Figure 5a). In particular, *TRBV7-8* was used frequently in adults and, to a lesser extent, in UCB precursors. More frequently, UCB precursors used *TRBV12-4* and *TRBV27*. In both populations, *TRBJ2-7* was preferred (Figure 5c). The CDR3β lengths were different with peaks at eight and nine residues for UCB precursors and adult memory cells, respectively (Figure 5e).

Finally, we undertook a more detailed analysis of repertoire architecture by calculating basic statistics for CDR3β length, *TRBV* and *TRBJ* gene usage (Supplementary Figure 1 and Supplementary Table 3). Distribution values for CDR3β length were similar across adult repertoires (median 12–14 amino acids, range 10–20) and naive precursor repertoires (median 12.5–13.5 amino acids, range 9–20). In contrast, there was a trend towards larger numbers of *TRBV* and *TRBJ* genes in the naive precursor populations. Collectively, these data suggest that naive CD8^+^ T-cell precursor repertoires are architecturally more diverse than the corresponding Ag-experienced memory repertoires.

### TCR bias in the naive Ag-specific T-cell precursor repertoire

TCR sequencing of Ag-specific cells revealed that UCB precursors exhibited biased chain architecture, often overlapping with the corresponding adult memory repertoires (Table 1). Identical (type III bias) and near-identical (type IV bias) TCR usage between individuals was observed within the A2-ELA specificity. Type III bias, whereby the same ‘public' TCR chain is used in multiple individuals,^19, 20^ was observed for clonotypes with TRAV12 transcripts. Similar repertoire biases were observed for A2-GIL. Notably, a public TRBV6-5 transcript present in A2-GIL-specific UCB precursors was curiously absent in adult memory. Highly homologous TRBV19 transcripts were present in both A2-GIL-specific UCB precursors and adult memory cells. Type III and type IV bias were also observed in A2-GLC-specific populations. Type IV bias and ‘TCR mosaicism', defined by alternate *TRBV* gene swapping around a common CDR3β core,^21^ were observed in the A2-NLV-specific repertoires, both within and between UCB precursors and adult memory cells.

## Discussion

In humans, around 170 αβ *TR* genes are recombined and somatically modified to create a circulating repertoire of ~10^12^ T cells bearing between 10^6^ and 10^11^ unique TCRs.^19^ Precisely what fraction of this grand repertoire is capable of recognizing any given peptide-major histocompatibility complex has been a lingering question for well over two decades. The recent arrival of multimer magnetic enrichment technology^3^ has provided a key to unlock the mysteries of the Ag-specific naive pool for the first time by using a direct and unmanipulated *ex vivo* approach. In the present study, we used UCB samples to obtain baseline frequency readings and found that Ag-specific T-cell precursors exist in the naive repertoire across a range between <1 and 352 cells per 100 000 CD8^+^ cells. These numbers are up to two orders of magnitude higher than those recorded previously in mice and human adults.^3, 10^ The frequency differences between UCB and adults can be explained relatively easily by the fact that, in adults, the naive compartment is generally smaller than memory, which directly impacts precursor calculations. However, why UCB precursor frequencies are higher than those found in newborn and/or pathogen-free mice is more difficult to explain. Humans have ~10 000-fold more T cells than mice.^19^ Assuming comparable TCR recognition degeneracy and proportionate repertoire diversity, humans should therefore encompass superior total antigenic coverage on a whole organism scale. However, when frequencies are assessed per 100 000 CD8^+^ T cells, differences between species should conceivably be harder to decipher.

Despite the genetic heterogeneity associated with outbred human populations, T-cell precursor frequencies were hierarchically clustered by Ag specificity as follows: A2-ELA>A2-GIL>A2-GLC>A2-NLV>B8-FLR>B7-TPR. The neonates used in this study were not related, typically sharing only the single HLA class I allele restricting the epitope of interest. This suggests that HLA haplotype does not greatly influence final precursor frequencies in the extrathymic pool. Given previous observations of a positive relationship between HLA haplotype and the magnitude of a memory T-cell response in virus-exposed individuals,^22^ these combined data suggest that HLA bias occurs after priming.

Naive precursors specific for A2-ELA were by far the most prevalent, peaking at one cell per 285 CD8^+^ thymic emigrants. What could explain these spectacularly high frequencies? Structural considerations are potentially informative in this setting. The TCR-A2-ELA ternary complex reveals TCR docking in an ‘α-centric' fashion via the *TRAV12* gene segment.^23^ Thus, it may be that large numbers of circulating clonotypes are capable of binding A2-ELA through minor modifications around a basic ‘*TRAV12* gene blueprint'. Additional evidence to support this idea comes from the observation of *TRAV12* gene-biased A2-ELA-reactive cells in HLA-A2^-^ individuals.^6^ An alternative but potentially synergistic explanation involves leaky tolerance as it has been shown that human medullary thymic epithelial cells mis-initiate transcription of the *Melan-A/MART-1* gene.^24^ High-frequency A2-GIL-specific cells can be explained similarly. These clonotypes bind in a 'β-centric' manner^25^, making heavy use of germline regions encoded by the *TRBV19* gene.^19^ Thus, akin to A2-ELA-specific clonotypes, there may be common A2-GIL-specific TCRs based around a '*TRBV19* gene blueprint'. Interestingly, we also identified a mysterious and equally large TRBV6-5^+^ population within the A2-GIL-specific naive repertoire. Some of the corresponding transcripts were found to be residue-identical between unrelated UCB donors, yet encoded via unique nucleotide sequences. Furthermore, we identified 'near-public' clonotypes within the TRBV6-5^+^ pool that differed at only one or two residues across the CDR3β loop. Although further data are required to assess the general applicability of this phenomenon, we found no evidence of these highly biased TRBV6-5^+^ TCRs in the adult memory compartment. In fact, we did not identify a single *TRBV6-5* gene transcript from our entire adult database of 631 A2-GIL-specific TCRs. This intriguing observation suggests that the naive Ag-specific repertoire is much more complex than previously thought, comprising multiple layers of publicity. Some public TCRs are preferred during Ag-driven selection while others are neglected. Exactly why this occurs is unknown, but the biology of these 'lost publics' is likely to be intriguing.

TCR recognition of A2-GLC, A2-NLV and B8-FLR has been studied at the atomic level by ourselves^26, 27^ and others.^28^ The ternary complexes reveal equally split footprints across the TCR α-chains and β-chains with the majority of bound Ag contacts focused on the CDR3 loops. These structural constraints may limit the final frequencies of cognate precursors in the naive pool. We observed considerable *TR* gene usage overlap between UCB precursors and adult memory when examining the A2-GLC, A2-NLV and B8-FLR datasets. However, in spite of these heavily biased naive repertoires, we failed to identify *bona fide* public receptors against these three Ags in UCB. One possible explanation for this unexpected finding is that public TCRs specific for these Ags may simply exist at low frequencies *in vivo*. This scenario would suggest that there is something inherently special about public TCRs, likely related to optimal Ag engagement, that allows the respective clonotypes to dominate the memory pool over decades of life.^29^

In agreement with our data, a recent study in mice found that: (i) naive T-cell precursors can cluster numerically by Ag specificity; (ii) T-cell precursor frequencies do not correlate with immunodominance hierarchies observed after pathogen encounter; (iii) public clonotypes are relatively rare in the precursor pool; and (iv) there is a distinct tapering of the TCR repertoire during transition to memory.^4^ Similar investigations in HIV-seropositive adults also failed to find a correlation between precursor frequency and immunodominance.^30^ These observations across species suggest that the above parameters of naive cellular immunity may be intrinsic to mammalian biology. In human adult peripheral blood, very recent enumerations have been placed at one Ag-specific cell per 1000–1000 000 CD8^+^ cells^7^ or one cell per 100 000–1000 000 CD4^+^ cells^10^ for both foreign and self-derived Ags. Additionally, for CD4^+^ precursors, memory-phenotype T cells were present in adults but not newborns.^10^ These findings may reflect T-cell activation via crossreactive ligands. Given this collective dataset, the enumerations of two Ag-specific precursor populations in our study can be compared with the corresponding specificities in adults.^7^ Such comparisons reveal that newborns have two-fold more A2-NLV-specific precursors and five-fold more A2-ELA-specific precursors.^7^ The cause of this enrichment is unknown, but it is thought that UCB T cells have a highly proliferative phenotype,^31^ which could conceivably increase precursor numbers. Likewise, intrinsic qualitative deficiencies in recruitment and/or division may thwart the mobilization of certain precursor clonotypes into memory.^32^

In summary, we explored the human newborn immune system to define and characterize baseline T-cell immunity against common viruses and immunogenic Ags. Despite considerable genetic heterogeneity, we found that T-cell precursor frequencies between neonates tended to cluster by Ag specificity. These frequency clusters varied by >300-fold, potentially as a consequence of disparate TCR docking modes. Molecular analysis of TCR usage revealed that the naive Ag-specific T-cell repertoire is typically broad and predictably structured between infants. Despite considerable overlap with the adult repertoire, however, it was clear that the naive repertoire is not an exact copy of memory. On the contrary, we found that the naive Ag-specific T-cell repertoire was complex, comprising interlayers of structurally biased receptors. Some public clonotypes were observed in the Ag-driven memory compartment, while others were apparently not selected. Further work is required to untangle these complex repertoire dynamics. Nonetheless, the observation that 'unexploited' Ag-specific clonotypes reside in the naive T-cell precursor pool opens up new possibilities for vaccine development. Indeed, only now are we beginning to understand the pathological consequences of 'clonotype choice', which can determine host outcome in the face of pathogen challenge.^33, 34, 35, 36^ Ultimately, the ability to target and amplify 'ideal' precursors from the naive pool, possibly through the use of TCR-optimized peptides to mobilize specific clonotypes,^37, 38, 39^ could be a potent advance for biomedicine.

## Methods

### Blood samples and processing

Density gradient centrifugation-enriched anonymized UCB lymphocytes were obtained from The Anthony Nolan Trust, Royal Free Hospital, Hampstead, UK. Healthy adult blood was obtained from donors at the Queensland Institute of Medical Research, Brisbane, Australia. Exposure to EBV and CMV was determined serologically via ELISA. Lymphocytes were isolated by Ficoll-Paque PLUS (GE Healthcare, Little Chalfont, UK) density gradient centrifugation and cryopreserved in R10 medium (RPMI-1640 containing 10% FBS) supplemented with 10% DMSO (Sigma-Aldrich, Gillingham, UK). For some UCB samples, cells were expanded by adding Human T-Activator CD3/CD28 Dynabeads (Life Technologies, Paisley, UK) as described previously.^40^ All samples were obtained with written consent and all protocols were approved by ethics committees at The Anthony Nolan Trust and the Queensland Institute of Medical Research.

### Antibodies, cell stains and dextramers

The following mAb conjugates were used at pre-titrated concentrations: (i) αCD3-APC-H7 (BD Biosciences, San Jose, CA, USA); (ii) αCD8-QD705, αCD14-Pacific Blue and αCD19-Pacific Blue (Life Technologies); (iii) αCD8-PE, αCD57-FITC and αCCR7-PE-Cy7 (BD Pharmingen, San Jose, CA, USA); and (iv) αCD27-PE-Cy5, αCD45RA-ECD and αCD45RO-ECD (Beckman Coulter, High Wycombe, UK). LIVE/DEAD Fixable Aqua and Violet Dead Cell Stain Kits (Life Technologies) were used to eliminate nonviable cells from the analysis. Peptide-major histocompatibility complex dextramers conjugated separately to APC and PE (Immudex, Copenhagen, Denmark) were used for magnetic enrichment.

### Dextramer-based magnetic enrichment and multiparametric flow cytometry

Cryopreserved UCB cells and Dynabead cultures were thawed into warm R10 medium. For each donor, 10^5^ cells were used to determine the percentage of CD8^+^ T cells in the sample. These cell aliquots were labeled with LIVE/DEAD Fixable Violet for 5 min on ice, then stained with αCD8-PE, αCD14-Pacific Blue and αCD19-Pacific Blue for a further 30 min on ice. Data were acquired using a FACSCanto II flow cytometer and analyzed with FACSDiva v6.0 software (BD Biosciences). The percentage of live CD14^-^ CD19^-^ CD8^+^ T cells in each sample was used in conjunction with total cell counts to determine the number of CD8^+^ T cells that underwent magnetic enrichment. To enrich for epitope-specific CD8^+^ T cells, samples were incubated in R10 medium containing 50 nM dasatinib (Axon Medchem, Groningen, Netherlands) for 30 min at 37 °C,^17^ then labeled with PE-conjugated dextramer for 30 min on ice. After washing in magnetic-activated cell sorting (MACS) buffer (degassed phosphate-buffered saline containing 0.5% bovine serum albumin and 2 mm EDTA; all Sigma-Aldrich), cells were resuspended in 80 μl MACS buffer with 20 μl αPE microbeads (Miltenyi Biotec, Bergisch Gladbach, Germany) per 10^7^ cells, incubated for 15 min on ice, washed again and resuspended in 2 ml MACS buffer. Each cell suspension was then passed through a separate magnetic separation (MS) column (Miltenyi Biotec). The columns were washed three times with 500 μl MACS buffer and removed from the magnetic field. Bound cells were eluted by pushing 2 ml MACS buffer through each column with a plunger. Enriched cells were centrifuged, labeled with LIVE/DEAD Fixable Aqua for 5 min on ice, then stained for 30 min on ice with a cocktail containing the following directly conjugated mAbs: αCD3-APC-H7, αCD8-QD705, αCD14-Pacific Blue, αCD19-Pacific Blue, αCD27-PE-Cy5, αCD45RA-ECD or αCD45RO-ECD, αCD57-FITC and αCCR7-PE-Cy7. After labeling, cells were washed with MACS buffer and acquired using a custom-modified FACSAria II flow cytometer (BD Biosciences). To isolate epitope-specific T cells, single lymphocytes were selected based on light scatter properties and dump^+^ (nonviable, CD14^+^ or CD19^+^) cells were gated out. From this population, up to 5000 dextramer^+^ CD8^+^ T cells were sorted at >98% purity directly into 1.5 ml screw-cap tubes (Sarstedt, Leicester, UK) containing 100 μl RNAlater (Life Technologies). The entire sample was acquired for each donor. Sorted cells in RNAlater were then centrifuged and frozen immediately at −80 °C for subsequent molecular analysis of TCR usage. Flow cytometric data were analyzed with FlowJo v7.6.5 software (TreeStar, Ashland, OR, USA). The frequency of dextramer^+^ CD8^+^ T cells in each sample was calculated by dividing the number of dextramer^+^ CD8^+^ T cells acquired on the FACSAria II by the total number of CD8^+^ T cells passed through the MS column. Frequencies were then converted to the number of dextramer^+^ CD8^+^ T cells per 100 000 CD8^+^ T cells.

### Molecular analysis of TCR usage

TCR clonotyping was performed as described previously.^18^ Briefly, all expressed *TRB* or *TRA* gene products were amplified without bias using a template-switch anchored PCR with reverse transcription incorporating a 3' TRB constant region primer (5′-TGCTTCTGATGGCTCAAACACAGCGACCT-3′) or a 3′ TRA constant region primer (5′-AATAGGCAGACAGACTTGTCACTGGA-3′). Amplicons were subcloned, sampled, Sanger sequenced and analyzed as described previously.^41^

## Figures and Tables

**Table 1:**
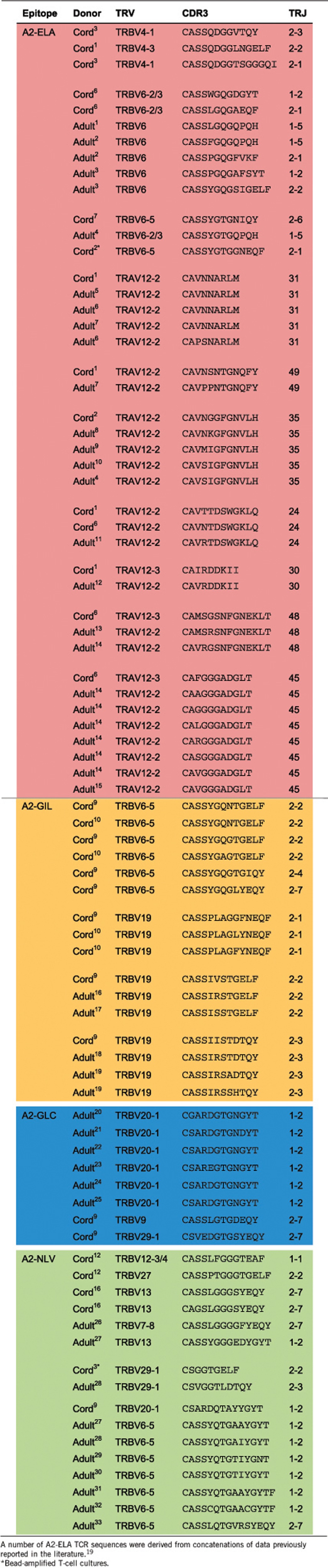
Ag-specific clonotype homology between naive and memory T cells
