# Supplementary figures and images for: Naive CD8+ T-cell precursors display structured TCR repertoires and composite antigen-driven selection dynamics
Source: Immunol Cell Biol. 2015 Mar 24;93(7):625–33. doi: 10.1038/icb.2015.17 (PMC4533101; doi:10.1038/icb.2015.17)

# Supplemental Figure 1

A

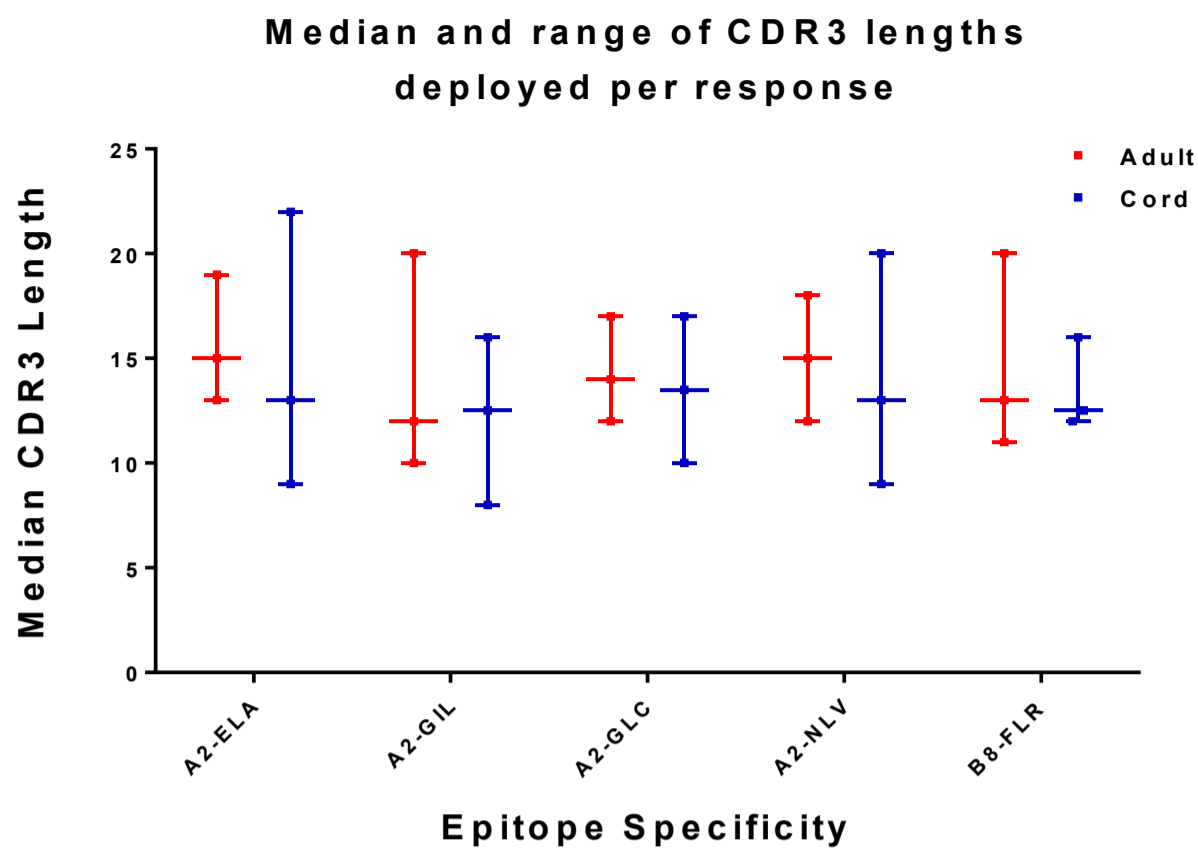

B

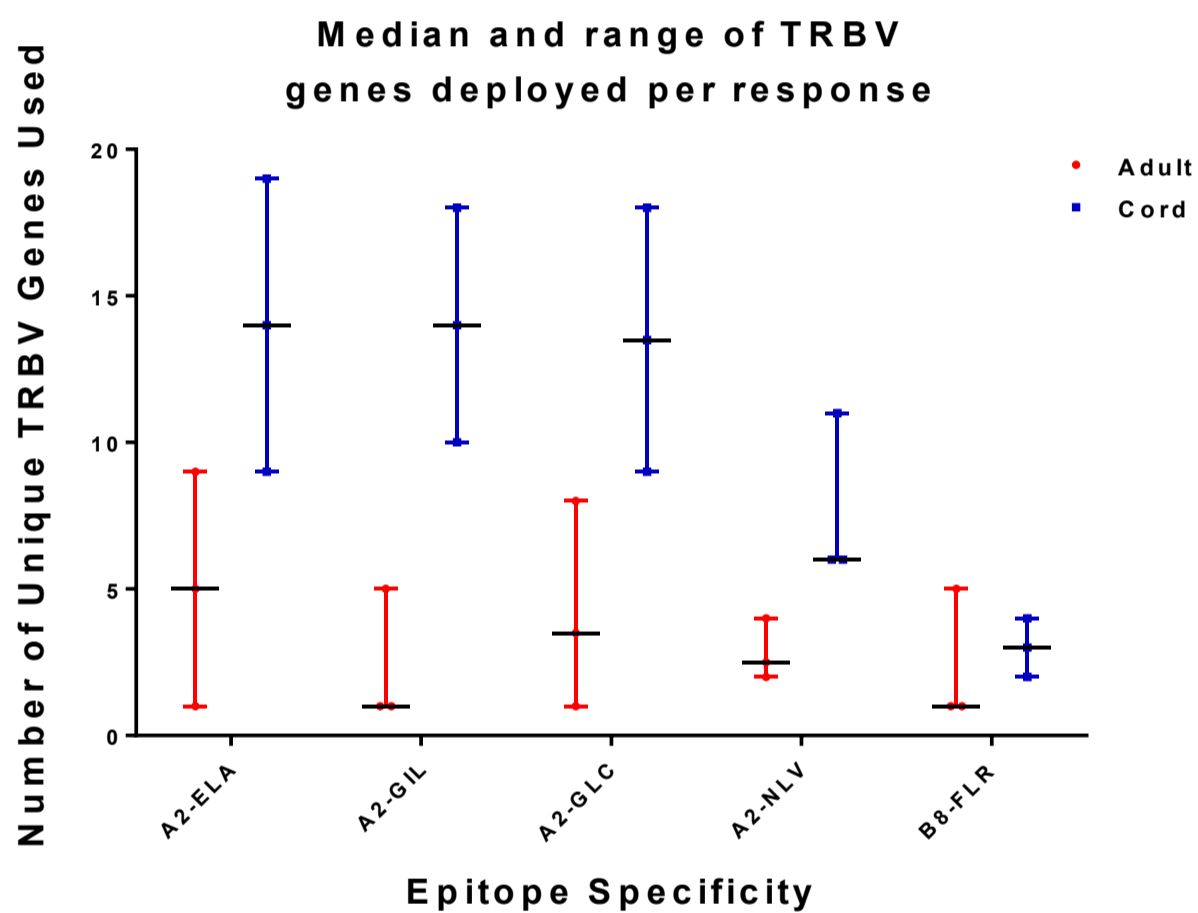

C

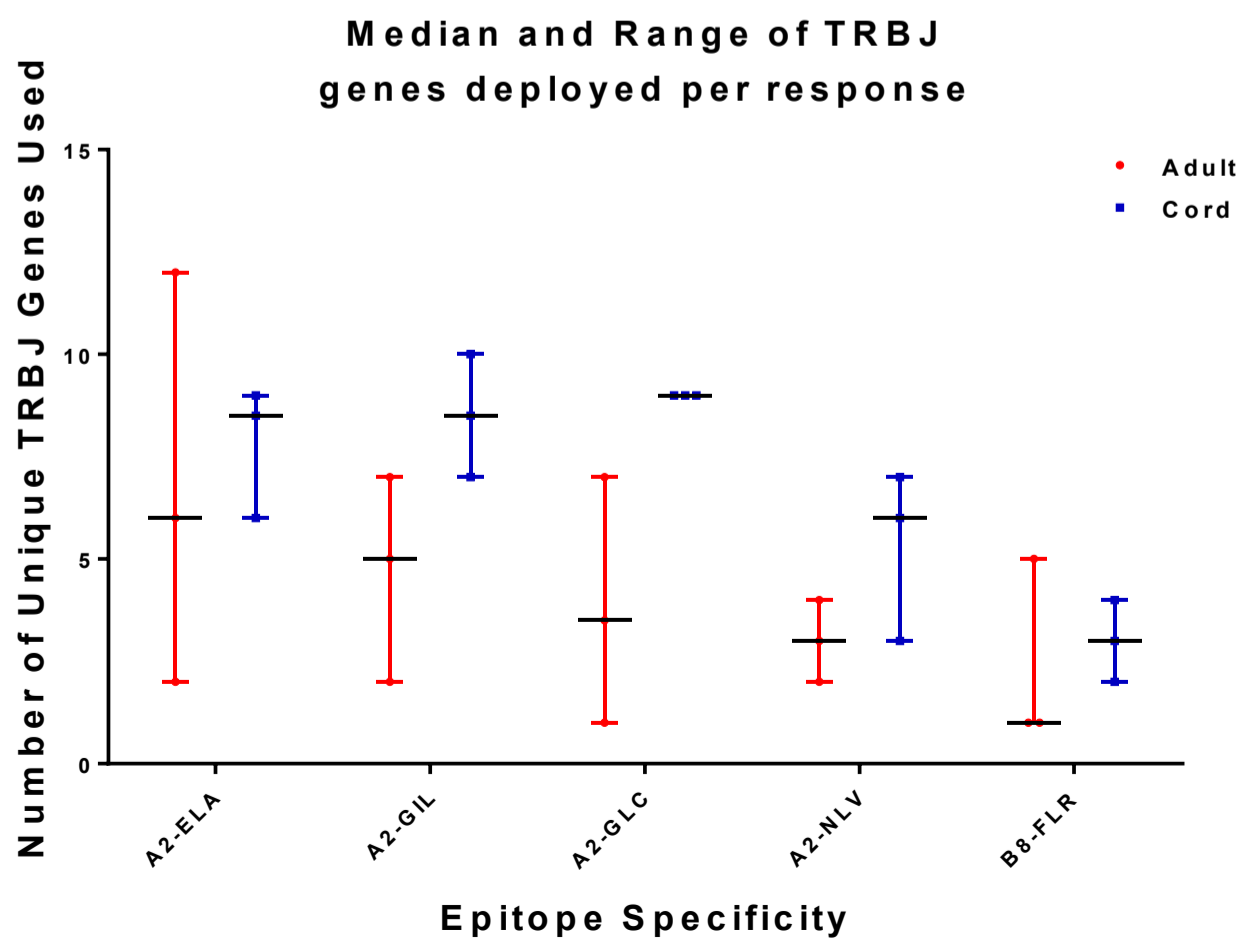

Supplement: Supplementary Figure 1 [file icb201517x1.pdf]
