# Supplementary material for: Naive CD8+ T-cell precursors display structured TCR repertoires and composite antigen-driven selection dynamics
Source: Immunol Cell Biol. 2015 Mar 24;93(7):625–33. doi: 10.1038/icb.2015.17 (PMC4533101; doi:10.1038/icb.2015.17)
Supplement: Supplementary Table S1 [file icb201517x2.pdf]

Supplemental Table S1. Ag-specific T cell precursor frequencies by donor.

Epitope	Sample	Total lymphocytes CD8+ (%)	Frequency of Dex+ (1 in _)	Dex+ Per 100,000 CD8+
A2-ELA	Cord[1]	13.9	285	352
	Cord[2]	16.4	377	266
	Cord[3]	14.1	456	220
	Cord[4]	25.6	397	252
	Cord[5]	20.5	468	214
	Cord[6]	25.4	481	208
	Cord[7]	19.5	494	202
	Cord[8]	12.2	833	120
A2-GIL	Cord[9]	16.9	6,579	15.2
	Cord[10]	24.7	19,204	5.2
	Cord[11]	7.74	1,495	66.9
	Cord[2]	12.5	6,708	14.9
	Cord[2*]	34	8,367	12
	Cord[3]	10.6	5,222	19.2
	Cord[3*]	46.8	5,978	16.7
	Cord[18]	21.2	22,552	4.4
	Cord[12]	27.6	85,157	1.2
	Cord[14]	17.6	44,675	2.2
A2-GLC	Cord[9]	21.3	4,147	24.1
	Cord[10]	19.1	2,881	34.7
	Cord[15]	16.5	32,322	3.1
	Cord[12]	33.3	63,599	1.6
	Cord[3]	14.6	34,111	2.9
	Cord[3*]	27.9	365,631	0.3
	Cord[1*]	25.8	87,606	1.1
	Cord[11]	6.99	15,571	6.4
	Cord[14]	12.1	5,198	19.2

Epitope	Sample	Total lymphocytes CD8+ (%)	Frequency of Dex+ (1 in _)	Dex+ Per 100,000 CD8+
A2-NLV	Cord[11]	14.6	34,208	2.9
	Cord[10]	12.8	40,550	2.5
	Cord[11]	6.99	15,084	6.6
	Cord[16]	11.2	28,103	3.6
	Cord[17]	8.74	14,576	6.9
	Cord[15]	13	53,189	1.9
	Cord[11]	20.4	31,824	3.1
	Cord[14]	12.1	25,555	3.9
	Cord[10*]	32.1	53,815	1.9
	Cord[3*]	24.2	31,360	3.2
B8-FLR	Cord[17]	11.8	21,111	4.7
	Cord[19]	26.5	57,596	1.7
	Cord[1]	14.1	42,523	2.4
	Cord[1*]	25.2	284,996	0.4
B7-TPR	Cord[3]	10.6	61,475	1.6
	Cord[1]	13.9	n/a	<1
A2-GLC	Adult[1]	21.4	472,031	0.2
	Adult[2]	20.7	1,698,642	0.1
A2-NLV	Adult[3]	15.7	47163	2.1
	Adult[4]	13.7	81883	1.2
	Adult[5]	15	127651	0.8
	Adult[6]	24.1	77235	1.3
	Adult[7]	19.7	76403	1.3

Color shading denotes Ag specificity as shown in Figure 1: self-derived A2-ELA (red), influenza-derived A2-GIL (gold), EBV-derived A2-GLC (dark blue), CMV-derived A2-NLV (green), EBV-derived B8-FLR (purple) and CMV-derived B7-TPR (light blue). Samples shown in the lower two panels were derived from EBV-seronegative (for A2-GLC) and CMV-seronegative (for A2-NLV) adults. *Bead-amplified T cell cultures.
