# Supplementary material for: Naive CD8+ T-cell precursors display structured TCR repertoires and composite antigen-driven selection dynamics
Source: Immunol Cell Biol. 2015 Mar 24;93(7):625–33. doi: 10.1038/icb.2015.17 (PMC4533101; doi:10.1038/icb.2015.17)
Supplement: Supplementary Table S2 [file icb201517x3.pdf]

Supplemental Table S2. Statistical analysis of frequency differences across Ag specificities.

A) Intra-antigen differences in precursor T cells (UCB)*

	A2-ELA	A2-GIL	A2-GLC	A2-NLV	B8-FLR	B7-TPR
**A2-ELA**		<0.0001	<0.0001	<0.0001	<0.0001	0.0016
**A2-GIL**	<0.0001		0.4802	0.0605	0.1936	0.3260
**A2-GLC**	<0.0001	0.4802		0.1091	0.2346	0.3497
**A2-NLV**	<0.0001	0.0605	0.1091		0.2216	0.1000
**B8-FLR**	<0.0001	0.1936	0.2346	0.2216		0.5041
**B7-TPR**	0.0016	0.3260	0.3497	0.1000	0.5041	

B) Intra-antigen differences in memory T cells (Adults)*

	A2-ELA	A2-GIL	A2-GLC	A2-NLV	B8-FLR	B7-TPR
**A2-ELA**		0.0814	0.4609	0.1068	0.1341	0.0243
**A2-GIL**	0.0814		0.0442	0.0155	0.0016	0.002
**A2-GLC**	0.4609	0.0442		0.1505	0.5511	0.0245
**A2-NLV**	0.1068	0.0155	0.1505		0.2691	0.1633
**B8-FLR**	0.1341	0.0016	0.5511	0.2691		0.0495
**B7-TPR**	0.0243	0.002	0.0245	0.1633	0.0495	

***P values determined using the unpaired t-test on T cell frequencies from Figure 1
