# Supplementary material for: Naive CD8+ T-cell precursors display structured TCR repertoires and composite antigen-driven selection dynamics
Source: Immunol Cell Biol. 2015 Mar 24;93(7):625–33. doi: 10.1038/icb.2015.17 (PMC4533101; doi:10.1038/icb.2015.17)
Supplement: Supplementary Table S3 [file icb201517x4.docx]

Supplemental Table 3

**CDR3 Loop Length**

|  | **Adult** | | **Cord** | |
| --- | --- | --- | --- | --- |
| **Epitope** | **Median Length** | **Range** | **Median Length** | **Range** |
| **A2-ELA** | 15 | 13-19 | 13 | 9-22 |
| **A2-GIL** | 12 | 10-20 | 12.5 | 8-16 |
| **A2-GLC** | 14 | 12-17 | 13.5 | 10-17 |
| **A2-NLV** | 15 | 12-18 | 13 | 9-20 |
| **B8-FLR** | 13 | 11-20 | 12.5 | 12-16 |

**Number of TRBV genes used**

|  | **Adult** | | **Cord** | |
| --- | --- | --- | --- | --- |
| **Epitope** | **TRBV Median** | **TRBV Range** | **TRBV Median** | **TRBV Range** |
| **A2-ELA** | 5 | 1-9 | 14 | 9-19 |
| **A2-GIL** | 1 | 1-5 | 14 | 10-18 |
| **A2-GLC** | 3.5 | 1-8 | 13.5 | 9-18 |
| **A2-NLV** | 2.5 | 2-4 | 6 | 6-11 |
| **B8-FLR** | 1 | 1-5 | 3 | 2-4 |

**Number of TRBJ genes used**

|  | **Adult** | | **Cord** | |
| --- | --- | --- | --- | --- |
| **Epitope** | **TRBJ Median** | **TRBJ Range** | **TRBJ Median** | **TRBJ Range** |
| **A2-ELA** | 6 | 2-12 | 8.5 | 6-9 |
| **A2-GIL** | 5 | 2-7 | 8.5 | 7-10 |
| **A2-GLC** | 3.5 | 1-7 | 9 | 9-9 |
| **A2-NLV** | 3 | 2-4 | 6 | 3-7 |
| **B8-FLR** | 1 | 1-5 | 3 | 2-4 |
